# Simulation-Based Training for Nursing Students to Improve Patient Safety: Systematic Review

**DOI:** 10.2196/87898

**Published:** 2026-05-26

**Authors:** Azucena González-Sanz, José Carlos López-García, Elena Sutil-Rodríguez, Raúl Juárez-Vela, José Ángel Santos-Sánchez, Noelia Navas-Echazarreta, Antonio Martínez-Sabater, Consuelo Sancho-Sanchez

**Affiliations:** 1 Primary Care Center Toro, Gerencia de Asistencia Sanitaria de Zamora (GAZSA), Zamora, Castille and León, Spain; 2 Primary Care Center Virgen Concha, Gerencia de Asistencia Sanitaria de Zamora (GAZSA), Zamora, Castille and León, Spain; 3 Primary Care Center Benavente Sur, Gerencia de Asistencia Sanitaria de Zamora, SACYL, Zamora, Castille and León, Spain; 4 CECAVIS Research Group, Instituto de Investigación Biosanitaria de León (IBIOLEON LEÓN), Leon, Castille and León, Spain; 5 Research Group in Care (GRUPAC), Pre-departmental Biomedical Sciences and Health Specialties Unit, Faculty of Health Sciences, Universidad of La Rioja, C/ Duquesa de la Victoria, 88, Logroño, La Rioja, 26004, Spain, 34 941299059; 6 Faculty of Medicine, Universidad de Salamanca, C. Alfonso X el Sabio, s/n, Salamanca, Castille and León, 37007, Spain; 7 Nursing Care and Education Research Group (GRIECE),Nursing Department, Universitat de València, Valencia, Valencia, Spain; 8 Care Research Group (INCLIVA), Hospital Clínico Universitario de Valencia, Valencia, Valencia, Spain

**Keywords:** nursing students, simulation training, patient safety, nursing, systematic review, PRISMA, Preferred Reporting Items for Systematic Reviews and Meta-Analyses

## Abstract

**Background:**

Patient safety is a fundamental pillar of health care quality. Simulation-based training provides a controlled environment for nursing students to develop safety competencies and error-recognition skills before clinical practice.

**Objective:**

This systematic review aimed to describe and characterize the simulation-based education features and modalities used to address patient safety outcomes in undergraduate nursing students, identifying the strategies that contribute to improvements in safety-related competencies.

**Methods:**

A systematic review was conducted following PRISMA (Preferred Reporting Items for Systematic Reviews and Meta-Analyses) guidelines across PubMed, Web of Science, Scopus, CINAHL, Cochrane, and Lilacs (2019‐2024). Inclusion criteria focused on original studies involving undergraduate nursing students and simulation interventions measuring patient safety outcomes. Studies in languages other than English, Spanish, or Portuguese were excluded. Two reviewers independently performed study selection and data extraction. Methodological quality was assessed using Joanna Briggs Institute tools, applying a 60% quality threshold for inclusion. Results were synthesized through a narrative approach.

**Results:**

A total of 20 studies from 12 countries were included. The methodological quality was high (n=14) and moderate (n=6). Findings revealed that high-fidelity simulation and virtual reality are the primary strategies used. Simulation proved effective in enhancing both technical skills (medication administration accuracy) and nontechnical skills (communication via SBAR [Situation, Background, Assessment, Recommendation] and ISBAR [Identification, Situation, Background, Assessment, Recommendation] tools, teamwork, and adverse event reporting). Key strategies contributing to safety included repetitive practice and interprofessional simulation, which significantly improved error detection and clinical judgment.

**Conclusions:**

Simulation is an essential pedagogical strategy for preparing nursing students to deliver safe care. Practical implications include the need to integrate structured simulation into nursing curricula to bridge the theory-practice gap. Future research should prioritize longitudinal designs to assess the retention of these safety skills in clinical settings and develop standardized metrics for measuring patient safety outcomes.

## Introduction

### Overview

Patient safety is a fundamental pillar of quality health care, aimed at minimizing risks and preventing harm during medical care [1]. According to the World Health Organization (WHO), this concept is defined as “the absence of avoidable harm to a patient and the reduction to an acceptable minimum of the risk of unnecessary harm associated with healthcare” [2]. Despite its importance, safety failures remain a critical global issue; more than 1 in 10 patients suffer harm during treatment, leading to more than 3 million deaths annually. Consequently, unsafe care must be recognized as both a major public health crisis and a significant economic burden [3].

The study of these failures gained international prominence following the landmark report “To Err is Human,” published in 2000 by the National Academy of Sciences. This study revealed that the cost of medical errors extends beyond economics, deeply affecting patient satisfaction, professional morale, and overall trust in the health system [4]. To address these challenges, a comprehensive monitoring approach is required. This involves the proactive identification of hazards, continuous process improvement, and the promotion of a safety culture that actively engages both health care professionals and patients [1].

Patient safety culture is a complex phenomenon in which values, attitudes, competencies, and behaviors influence how health care professionals perceive and manage safety risks [2]. A comprehensive framework for patient safety culture consists of leadership, teamwork, evidence-based practice, communication, learning, just culture, and patient-centered care [5]. This concept is integrated with WHO resolution WHA72.6 [6], which prioritizes concrete measures to prevent avoidable harm in health care, such as strategies for safe medication, error-free surgery, infection control, sepsis management, reliable diagnostics, and adequate hygiene in facilities. This multidimensional framework highlights the systemic nature of safety culture and the central role of leadership and communication in preventing adverse events.

The occurrence of safety incidents is rarely the result of a single individual’s actions but rather the culmination of various systemic factors [4]. These include rapid technological advances, increasingly complex care processes, inadequate policies, and an aging population with multiple chronic comorbidities [2]. Because incidents often stem from multiple overlapping causes, focusing on individual blame fails to address the underlying systemic issues, making it likely that the same errors will recur [2,4]. To mitigate these risks, the Global Patient Safety Action Plan 2021‐2030 outlines strategic activities designed to identify, evaluate, and manage risks throughout the health care continuum, with the ultimate goal of preventing harm or minimizing its impact [7].

Training and education in safe practices are essential to maintaining and improving standards of care and ensuring that health systems can adapt and respond to emerging challenges in health care [1]. In this context, simulation, defined as any technique that extends or replaces real-world experiences to promote reflective learning, has established itself as a key strategy for improving research on patient safety incidents [8].

Clinical simulation has proven effective as a teaching strategy for developing both technical and nontechnical skills, including communication, teamwork, leadership, and critical thinking [9,10]. This approach allows for the creation of structured, meaningful, and reflective learning environments that facilitate the effective and safe resolution of complex situations, in line with the competencies to be acquired [8-10].

Clinical scenarios form the basis of clinical simulation, as they allow learning experiences to be structured with different levels of complexity in line with training objectives [11]. A key element in their design is fidelity, understood as the degree to which the simulation reproduces the conditions of actual clinical practice [12], which can be classified as low, medium, or high depending on the resources used and the educational goals. Low-fidelity simulation typically focuses on the acquisition of basic technical skills using task trainers, simple manikins, or case studies, while medium and high levels incorporate more complex scenarios and greater realism. Similarly, the simulation modality refers to the format used to develop the training experience. According to the Healthcare Simulation Dictionary, these modalities can include role-playing, virtual or online simulations, task trainers, high-tech mannequins, or immersive simulations with standardized patients (SPs) or trained actors [12]. Thus, the combination of modality and fidelity level allows simulation to be adapted to different educational contexts and clinical competencies [11]. Immersive simulation, particularly when based on realistic clinical scenarios, offers strong opportunities to develop competencies and promote changes in thinking and practice. Its effectiveness is enhanced when followed by structured debriefing, enabling reflective analysis and identification of improvement strategies [9].

Nursing professionals, from their formative stage, need to be actively trained to provide safe care, and effective communication is essential to reduce health errors [13,14]. A culture of open communication facilitates enhanced team interactions and equips students with efficacious strategies to deploy in their practice, thereby fostering critical thinking and emergency management skills. The formation of effective interprofessional teams through simulation-based learning has been linked to improved patient outcomes, a reduction in medical errors, and the delivery of high-quality care [15]. The students have acquired knowledge and have been instructed in the guidelines for conducting practices more safely. However, they encounter obstacles when attempting to communicate and report errors, as they perceive themselves to have a lower status and are sometimes fearful of the potential consequences [16].

A coalition of international scientific societies with expertise in simulation has issued a call for the integration of simulation-based learning into the curricula of undergraduate and postgraduate programs. This initiative is designed to foster a culture of safety [17]. It is also imperative that the simulation be designed in such a way that it does not compromise the psychological and physical integrity of the participants, while also ensuring the safety of the training process [18].

Previous reviews on educational interventions in patient safety highlight considerable methodological heterogeneity and limited evidence regarding the specific impact of simulation [19]. One review found that although patient safety competence was frequently assessed, only 2 studies used simulation, and none examined behavioral changes, limiting conclusions about real clinical impact [18]. A published protocol targeting nursing students also considers simulation among various teaching methods, but anticipates substantial variability that may hinder conclusions about its differential effectiveness [20].

Another systematic review reported improvements in knowledge, clinical skills, and confidence through simulation-based learning; however, it included varying fidelity levels and did not specifically address patient safety outcomes, limiting applicability to this domain [21]. Similarly, quantitative syntheses confirm the benefits of simulation but do not analyze its transfer to patient safety in real health care contexts [22]. A comparison between SPs and role-playing showed improvements in communication skills, yet without linking findings to objective patient safety indicators [23]. Finally, an exploratory review associated simulation with greater competence and confidence but did not differentiate between levels of evidence or modalities, limiting the strength of its recommendations [24].

Overall, despite suggested benefits, the literature shows methodological heterogeneity, limited analysis of behavioral outcomes, and insufficient connection to specific patient safety indicators, raising the question of the actual effect of simulation-based learning on patient safety outcomes in nursing students.

### Objective

Since 2020, with the closure of in-person training activities and the impossibility of clinical practices, justified by immediate public health needs: preventing the transmission of infections, facilitating social distancing, and responding to government orders, the need for education and training that adapts to the new demands of the health system has become evident [25,26]. The use of simulation methodology in the education of nursing students can be a strategy that improves the application of the theoretical concepts learned and clinical practice, to provide safe and quality care. The objectives of this systematic review were (1) to determine the effect of simulation-based education on patient safety outcomes in nursing students, (2) to identify the aspects related to patient safety that have been the subject of the use of simulation, and (3) to describe and characterize simulation-based education features and modalities used to address patient safety outcomes in undergraduate nursing students.

## Methods

### Design

A systematic review was carried out during the months of February and March 2024, following the checklist of the PRISMA (Preferred Reporting Items for Systematic Reviews and Meta-Analyses) statement [27]. The review followed the PRISMA 2020 guidelines and was designed and presented as a systematic review rather than an exploratory review, to accurately determine the effect of simulation-based education on patient safety outcomes, establishing strict predefined eligibility criteria, quality assessment, and structured synthesis of evidence (Checklist 1).

### Search Strategy

A systematic search was carried out from 2019 to 2024 in the databases: PubMed, Web of Science, Scopus, CINAHL, Cochrane, and Lilacs. These databases were selected to ensure broad coverage of biomedical, nursing, educational, and multidisciplinary research, as well as to minimize publication and indexing bias. To guide the search, we started from the PIO (Population, Intervention, Outcomes) question (Table 1).

The terms were adapted to the controlled language of the MeSH (Medical Subject Headings) and Descriptors in Health Sciences, in addition to CINAHL subject headings (Table 2).

**Table 1.** Keywords PIO^a^.

PIO question	Keywords	MeSH^b^	DeCS^c^
Population	Nursing students	Nursing students	Estudiantes de enfermería
Intervention	Training through simulation techniques	Simulation training	Simulación
Outcomes	Improving patient safety	Patient safety	Seguridad del paciente

a PIO: Population, Intervention, Outcomes.

b MeSH: Medical Subject Headings.

c DeCS: Descriptors in Health Sciences in Spanish.

**Table 2.** Search strategy.

Base	Search strategy	Number of articles
PubMed	((“education, nursing”[MeSH Terms] OR (“education”[All Fields] AND “nursing”[All Fields]) OR “nursing education”[All Fields] OR “education nursing”[All Fields] OR (“nursing education research”[MeSH Terms] OR (“nursing”[All Fields] AND “education”[All Fields] AND “research”[All Fields]) OR “nursing education research”[All Fields])) AND "2019/02/27 00:00”:“3000/01/01 05:00”[Date - Publication] AND ((“students, nursing”[MeSH Terms] OR (“students”[All Fields] AND “nursing”[All Fields]) OR “nursing students”[All Fields] OR (“nursing”[All Fields] AND “students”[All Fields])) AND (“simulation training”[MeSH Terms] OR (“simulation”[All Fields] AND “training”[All Fields]) OR “simulation training”[All Fields]) AND (“patient safety”[MeSH Terms] OR (“patient”[All Fields] AND “safety”[All Fields]) OR “patient safety”[All Fields]) AND "2019/02/27 00:00”:“3000/01/01 05:00”[Date - Publication] AND "2019/02/27 00:00”:“3000/01/01 05:00”[Date - Publication] AND "2019/02/27 00:00”:“3000/01/01 05:00”[Date - Publication])) AND (y_5[Filter])	92
Scopus	(TITLE-ABS-KEY (nursing AND students) AND TITLE-ABS-KEY (simulation AND training) AND TITLE-ABS-KEY (nursing AND education) AND TITLE-ABS-KEY (patient AND safety)) AND PUBYEAR >2018	109
WoS^a^	(((TS=(nursing students)) AND TS=(simulation training)) AND TS=(nursing education)) AND TS=(patient safety) Last 5 years	136
LILACS	(estudiantes de enfermería) AND (simulación) AND (educación en enfermería) AND (seguridad del paciente) AND (year_cluster:[2019 TO 2024])	14
CINAHL	nursing students AND nursing education AND (simulation training or simulation education or simulation learning) AND (patient safety or patient outcomes or quality of care) Limiters - Publication Date: 20190101‐20241231	133
Cochrane	(*simulation training) AND (nursing students):ti,ab,kw AND (patient safety):ti,ab,kw (Word variations have been searched) con año de publicación de 2019 hasta 2024, fecha de publicación en la Biblioteca Cochrane Entre Jan 2019 y Feb 2024, en Ensayos	32

a WoS: Web of Science.

### Inclusion and Exclusion Criteria

The inclusion criteria were articles written in English, Spanish, and Portuguese, which were primary studies on the use of simulation in the undergraduate stages of nursing to maintain patient safety, such as communication, teamwork, medication safety, clinical performance linked to safety, without differentiating the type of simulator used. Although initially it was decided to include only nursing students, it was later decided to also include studies involving medical students in the case of communication or teamwork.

Exclusion criteria were considered qualitative studies, reviews, editorials, personal experiences, and those quantitative articles that were not peer reviewed, studies that dealt exclusively with student satisfaction and perception with the simulation methodology, studies in which among the participants there were already graduated professionals, and those in which at least one of the objectives did not deal with safety, studies on the use of simulators that were specific for learning specialized care, studies referring to the design of simulation scenarios or the development of modifications of educational strategies related to simulation.

### Article Selection, Methodological Quality Assessment, and Risk of Bias

The articles obtained were exported to the Zotero bibliographic manager. After removing duplicates, articles were selected by title and abstract during online sessions by 2 researchers. When discrepancies arose, another revisor was contacted for a final decision. Full texts of the remaining articles were obtained to assess eligibility. Two reviewers thoroughly assessed the full texts to determine study inclusion, resolving discrepancies through discussion and, when necessary, with the involvement of a third reviewer. Two reviewers determined that articles met eligibility criteria. References from secondary articles, such as reviews and scoping reviews, were also reviewed to assess their potential inclusion of those articles that met the objectives of this study. Those selected underwent a quality evaluation by 2 authors independently using the Joanna Briggs Institute (JBI) Critical Appraisal Tools [28] according to study design (the checklist for randomized controlled trials [RCTs] [29], the checklist for quasi-experimental studies [30], and the checklist for analytical cross-sectional studies [31]).

The methodological quality of all included studies was assessed independently by 2 reviewers using the JBI Critical Appraisal Tools according to the study design: the checklist for RCTs, quasi-experimental studies, and analytical cross-sectional studies. Discrepancies were resolved by consensus with a third reviewer. Studies meeting ≥60% of the applicable JBI criteria were included in the final synthesis. Considering that the JBI critical appraisal tools do not establish universal cutoff points for classifying methodological quality, leaving this decision to the discretion of review teams, a 60% threshold was applied as a pragmatic criterion to balance the inclusion of methodologically acceptable studies while avoiding overly restrictive exclusions, thereby ensuring the availability of sufficient evidence for analysis [28].

### Data Extraction and Synthesis

The authors independently extracted data from the studies into a Microsoft Excel spreadsheet. The sheet recorded data on the author, year of publication, country, age of participants, type of study, objective, scales and instruments used, results and conclusions, limitations, safety aspects covered, type of simulation, types of interventions performed, and simulated cases (Table S7 in Multimedia Appendix 1 [32-51] and Table S8 in Multimedia Appendix 2 [32-51]). Given the diversity among the included studies, clinical and methodological heterogeneity (diverse domains, modalities, and durations), noncomparable instruments, and incomplete statistical reporting (missing effect sizes and variances), a narrative synthesis was conducted, guided by the principles of thematic content analysis.

## Results

### Study Selection

**Figure 1.**
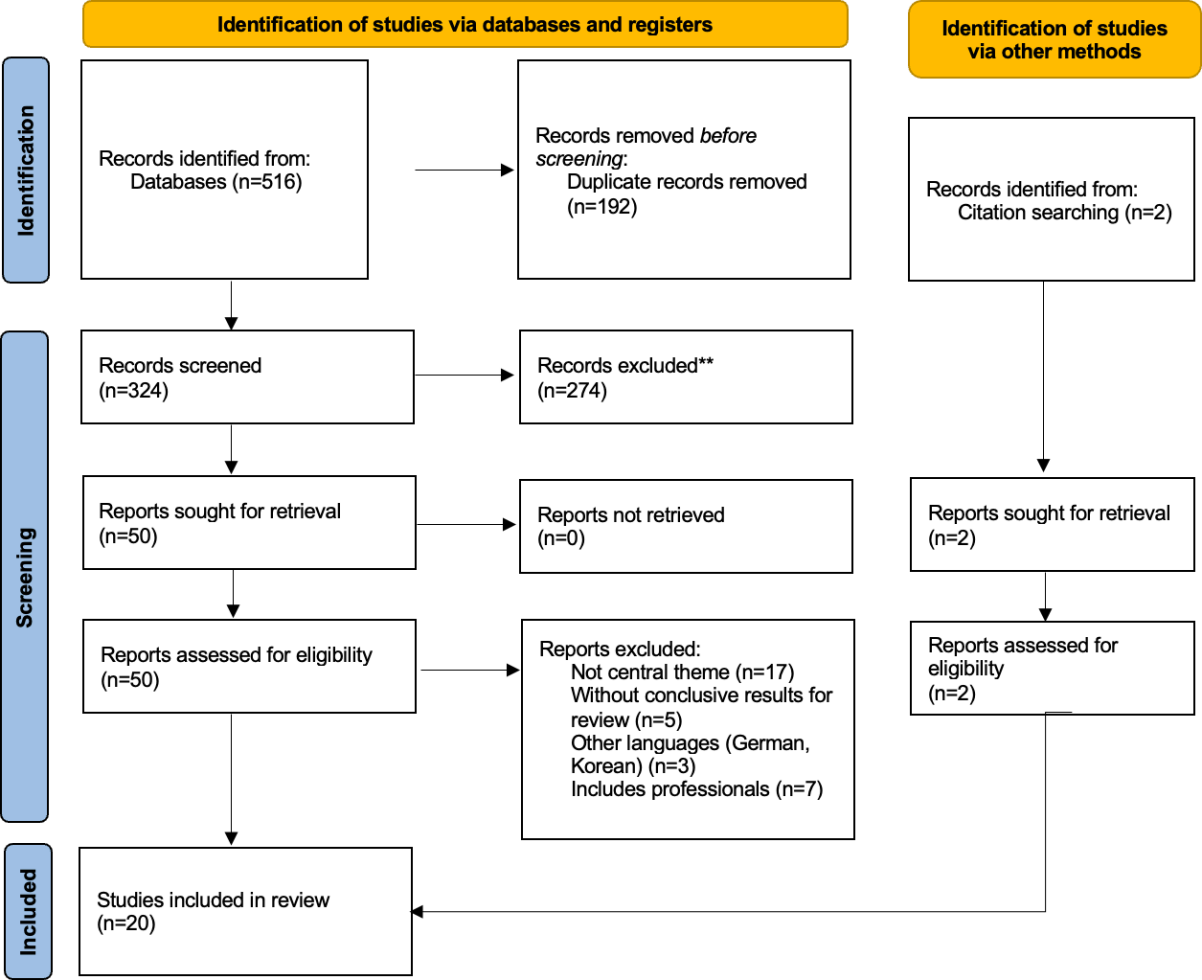
PRISMA (Preferred Reporting Items for Systematic Reviews and Meta-Analyses) 2020 flow diagram of study selection.

The selection process is shown in Figure 1.

### Characteristics of Included Studies

A total of 20 studies [32-51] met the inclusion criteria for this review. The primary characteristics of these studies are detailed in Table 3. The included articles were published between 2019 and 2024. The country with the highest number of studies was Korea with 6 studies [32,34,36,37,41,48], followed by the United States [32,40,45] and China [39,43,49], with 3 studies each. Spain [42,50] and Taiwan [46,51] contributed 2 studies each. Germany [35], Ireland [47], and Indonesia [38] contributed 1 study each. One study was led by an international team (Canada, England, Scotland, and Australia) [44]. For the purpose of counting, each of the 20 studies [32-51] was assigned to the country of the coordinating institution, and the multicountry study was considered as a single study with international participation.

**Table 3.** Characteristics of included studies.

Author (year), country	Participants	Type of study and assignment	Objectives	Data collection scales and instruments	Results and conclusions	Limitations
Breen et al [47] (2019), Ireland	Total 90: 45 nursing students 3rd course, 45 last-year medical students. Year: 2016.	ECA 3 groups: e-learning (E)^a^, E+standard simulation (E+S)^b^, competency-based progression simulation and e-learning (E+PBP)^c^. Randomized	To determine the effectiveness of a competency-based training (PBP)^d^ for clinical communication.	National Early Warning Score (NEWS)^e^ based on the ISBAR^f^ tool	Communication competence was achieved by 2/29 (7%) in E, 3/23 (13%) in E+S, and 15/25 (60%) in competency-based progression. Competency-based training was significantly more effective (*P*<.001)	Single center; undergraduate sample; short training duration
Son and Kim [36] (2019), Korea	98 Nursing students. CG^g^: 41, IG^h^: 57 Course 3rd Year: 2017 Age: 22‐23 Gender: Women	Quasi-experimental. Pretest-posttest design of nonequivalent control group. Convenience sampling	Effectiveness of communication education based on SEGUE^i^ between students and patients.	Communication competence and effectiveness measured by a self-report questionnaire and by teacher and standardized patient ratings of the students	Intervention students showed significantly greater improvements in communication competence and efficacy than controls (In terms of performance, as assessed by lecturers and students, the *P* value is <.001, the standardized patient score is 0.042, and the communication effectiveness score is 0.004).	Single institution; small control group
Jeong and Kim [37] (2020), Korea	54 nursing students. IG: 26, CG: 28 Grade: 3rd Year: 2018 Age: 20‐40 average 23 Gender: Women, only 1 man	Clinical trial (pre-post). Simple blind	Develop a fall simulation program using the SBAR^j^ communication technique	Knowledge of falls and attitude toward falls scale, post-fall evaluation protocol (AHRQ^k^). GICC-15^l^ and Dunsford adapted structured communication	SBAR-based simulation significantly improved structured fall-related communication compared with controls.	No long-term follow-up; single-blind design
Liaw et al [49] (2020), China	120 medical and nursing students. IG: 60, CG: 60 Grade: 3rd-4th year. Year: 2018. Mean age 22.17 (SD 2.07) years. Gender: Women (81, 67.5%)	RCT^m^ (pretest-posttest) Randomized	To evaluate a team training program using VR^n^ versus conventional live simulations on the performance of communication skills and teamwork	ATHCT^o^ and ISVS^p^. Baseline, posttest, and 2 months after	Both groups improved teamwork attitudes; VR training was noninferior with higher follow-up ISVS.	Self-reported outcomes; single-center design
Sanko and McKay [33] (2020), United States	231 nursing students from 2016 (CG: 68), 2017 (IG: 85), and 2018 (CG: 78) patient safety courses.	Clinical trial (2 cohorts years different intervention and control groups). Convenience sampling	To assess whether exposure to simulation scenarios to enhance systematic thinking influenced adverse event reporting and the type	Simulated AERS^q^. System Thinking Scale (STS)^r^	Systems thinking scores increased significantly after simulation, and adverse event reporting improved (*P*<.001).	Single-site sample; simulated reporting system
Lee and Kim [34] (2020), Korea	194 nursing students. 47 teams (4 and 3 components) Course: higher level. Year: 2015‐2016	Prospective observational convenience sampling	To examine the relationships between nursing students’ team task performance and SBAR-R^s^ communication.	Measurement of team task performance and communication using developed checklists based on SBAR-R	Higher SBAR-R scores were significantly associated with better team task performance (*P*=.004).	Single university; limited scope
Wai et al [48] (2021), Korea	46 students: 19 medical, 27 nursing. Interprofessional teams. Grade: Final year medicine (5th), 3rd-4th year nursing. Gender: 63% female students.	Mixed methods convenience sampling	To compare the effectiveness of combined classroom plus clinical simulation versus clinical simulation alone on attitudes, perceptions, and teamwork performance.	HFAS^t^ Survey. Teamwork performance using TBL-SAI^u^ focus group interview	Both groups improved teamwork attitudes (*P*=.04) with no added benefit from classroom teaching	Small sample; limited power
Musharyanti et al [38] (2021), Indonesia	95 nursing students IG:55. CG: 40.	Quasi-experimental. Nonequivalent randomized control group.	To compare drug safety knowledge and skills after safety training with the 4C/ID^v^ teaching method.	MCQs^w^ and 2 checklists developed ad hoc on patient safety and medication administration	Intervention students achieved significantly higher safety knowledge and skills than controls (*P*<.001).	No baseline testing
Du et al [39] (2021), China	47 nursing students.CG:21, IG:26 Grade: 2nd Year 2019. Age 17‐27 Gender: 44 women -3 men	Controlled trial. Simple blind.	To assess the risk of pressure ulcer development in 3 different scenarios	OSCE^x^ adapted to different scenarios Pressure Ulcer Knowledge Assessment Tool (PUKAT 2.0)^y^	OSCE-based simulation significantly improved assessment performance (*P*<.001).	Small sample. Single center
Lee and Lim [32] (2021), Korea	30 nursing students. Grade: Final year. Year: 2018. Average age 22.17 Gender: women	Quasi-experimental (pre-post). Convenience sampling	Develop, implement, and verify the effectiveness of a simulation-based handover education program.	Communication tool adapted from SBAR. Knowledge questionnaire developed by authors. Self-efficacy (adapted questionnaire).PASS-BAR^z^	Simulation significantly improved handover knowledge, self-efficacy, and performance (*P*≤.001).	No control group; all-female sample
Craig et al [40] (2021), United States	83 nursing students. CG:35. IG:45 Grade: 3rd.	Quasi-experimental. Convenience sampling	To examine the effects of an educational strategy using a simulation program on medication management.	MSKA^aa^ and MSCEC^ab^	Simulation significantly improved medication administration skills (*P*<.001).	Single-site study
Raurell-Torreda et al [50] (2021), Spain	93 nursing students. IG: 48, CG:45 Course: 3rd, 1 medical student in 5th year. Age: CG: 22.3±5.2- IG: 23.3±6.8 Gender: 78.5% women	Clinical Trial Randomized	Evaluate the impact of SBAR training on interprofessional teamwork skills (role-related and communication) and nontechnical skills.	KidSIM-TPS^ac^ and CSET^ad^ (nontechnical skills)	Intervention students showed significant improvements in teamwork behaviors (*P*≤.004).	Partial implementation
Park and Kim [41] (2021), Korea	91 nursing students. IG: 47, CG: 44. Final year of nursing Academic year 2018/19. Age: IG 22.59±1.23, CG 22.86±1.39 Gender:+77% women	Clinical Trial Randomized	Analyze the impact of simulated patient deterioration on situational awareness and patient safety competence-attitude.	SAGAT^ae^ modified; PSCSE^af^ modified.	Simulation significantly improved situational awareness and safety attitudes (*P*<.001).	Single university
Chen et al [51] 2022, Taiwan	54 students: 18 medical students and 36 nursing students Grade: 4th year nursing, 5th year medicine Year: 2019 Gender: 70% Women	Mixed methods Randomized	Determine the importance of interprofessional training on competence, teamwork attitudes, and safety.	MTP^ag^, TBP^ah^, TA^ai^, and PSA^aj^	Both groups achieved comparable competence gains; qualitative data supported learning benefits	Small sample
Pol-Castañeda et al [42] (2022), Spain	179 nursing students. Grade: 2nd Academic year 2018/19 Age: 60% between 18‐25 Gender: 89% women	Mixed methods Convenience sampling	To assess the acquisition of skills in safe medication administration by nursing students	Questionnaire. adapted from the. MASAT^ak^	Simulation improved most medication skills except documentation.	Assessment variability
Goldsworthy et al (2022), Canada, England, Scotland, and Australia [44]	88 nursing students 5 diverse university sites in 4 countries Grade: 3rd-4th Pandemic year 2020?	Quasi-experimental Convenience sampling	Explore the impact of a virtual simulation on to recognize and respond to a rapidly deteriorating patient.	10-item Clinical Self-Efficacy Survey designed 20-item multiple-choice test on evidence-based practice	Virtual simulation significantly improved knowledge scores (*P*=.001).	Nonrandomized design
Li et al [43] (2023), China	205 nursing students.IG:103, CG: 102. Grade: 2nd Academic year 2020/21. Mean age CG: 19.65 (SD 0.75) years, IG:19.78 (SD 0.77) years. Gender: women 87%‐89%	Quasi-experimental (2-tailed) Convenience sampling	Exploring the effects of an online course (SPOC)^al^ combined with simulation-based training in a patient safety education program.	PSCSE	Patient safety competence scores were significantly higher in intervention students (*P*<.001).	Single-center study
Haerlin et al [45] (2023), United States	193 nursing students: Group Clinical experience: 51, Group Mannequins:44, Group VR: 57. Academic year 2021/22. Age: 19-53 (median 21). Gender: 81.6% female	Quasi-experimental Convenience sampling	Compare differences in learning and practice in patient care as a function of the learner’s training experience.	Competency by CCEI^am^ and LCJR^an^. Clinical Learning CLECS^ao^ 2.0	Manikin-based simulation produced equal or superior outcomes compared with other modalities.	Regional sample
Chou et al [46] (2024), Taiwan	84 nursing students IG: 42 and CG: 42. Grade: 2nd Year: 2022. Mean age 20.3 (SD 0.46) years. Gender: 80% women	Clinical Trial Randomized	To examine the effectiveness of a VR communication simulation in the acquisition of communication skills in the fundamentals of nursing practice.	Kalamazoo Consensus Statement Essential Elements Communication Checklist, “Communication Self-Assessment Scale” modified, Development and Testing of a Perceived Stress Scale for Nursing Students in Clinical Practice and a learning satisfaction	Kalamazoo Consensus Statement Essential Elements Communication Checklist, “Communication Self-Assessment Scale” modified, Development and Testing of a Perceived Stress Scale for Nursing Students in Clinical Practice and a learning satisfaction	Short intervention; self-report bias
Heier et al [35] (2024), Germany	221 Students, 154 medical and 67 nursing students (IG: 66 medical, 28 nursing/CG: 88 medical, 39 nursing) Course: 3rd year medical and 1st-2nd year nursing students. Year: October 2021-March 2023. Mean age 24 (SD 3.9) years. Gender: 51.13% women	Mixed methods Convenience sampling	Develop joint communication skills training for nursing and medical students in professional error communication.	Adaptation of G-IPAS^ap^ “Teamwork,Roles and Responsibilities,” “Patient- centeredness” and a self-developed interprofessional error communication scale	Adaptation of G-IPAS “Teamwork,Roles and Responsibilities,” “Patient- centeredness” and a self-developed interprofessional error communication scale	Nonrandomized design

a E: e-learning.

b E+S: e-learning plus standard simulation group.

c E+PBP: e-learning plus competency-based progression simulation group.

d PBP: progression-based performance/competency-based progression.

e NEWS: National Early Warning Score.

f ISBAR: Identification, Situation, Background, Assessment, Recommendation.

g CG: control group.

h IG: intervention group.

i SEGUE: Set the stage, Elicit information, Give information, Understand the patient’s perspective, End the encounter.

j SBAR: Situation, Background, Assessment, Recommendation.

k AHRQ: Agency for Healthcare Research and Quality.

l GICC-15: General Interpersonal Communication Competency (15 items).

m RCT: randomized controlled trial.

n VR: virtual reality.

o ATHCT: Attitudes Toward Interprofessional Health Care Team.

p ISVS: Interprofessional Socialization and Valuing Scale.

q AERS: Adverse Event Reporting System.

r STS: Systems Thinking Scale.

s SBAR-R: Situation, Background, Assessment, Recommendation, Read-back.

t HFAS: Human Factors Attitude Survey.

u TBL-SAI: Team-Based Learning Student Assessment Instrument.

v 4C/ID: Four-Component Instructional Design.

w MCQ: multiple choice questionnaire.

x OSCE: Objective Structured Clinical Examination.

y PUKAT: Pressure Ulcer Knowledge Assessment Tool.

z PASS-BAR: Patient Safety Screen-Based Assessment Record (handover tool).

aa MSKA: Medication Safety Knowledge Assessment.

ab MSCEC: Medication Safety Critical Elements Checklist.

ac KidSIM-TPS: KidSIM -Program Team Performance Scale.

ad CSET: Clinical Simulation Evaluation Tool.

ae SAGAT: Situational Awareness Global Assessment Technique.

af PSCSE: Patient Safety Competency Self-Evaluation.

ag MTP: Medical Task Performance.

ah TBP: Team Behavior Performance.

ai TA: Teamwork Attitude.

aj PSA: Patient Safety Attitude.

ak MASAT: Medication Administration Safety Assessment Tool.

al SPOC: Small Private Online Course.

am CCEI: Creighton Competency Evaluation Instrument.

an LCJR: Lasater Clinical Judgment Rubric.

ao CLECS: Clinical Learning Environment Comparison Survey.

ap G-IPAS: German Interprofessional Attitudes Scale.

The study designs varied, encompassing 8 RCTs [33,37,39,41,46,47,49,50], 11 quasi-gstudies [32,35,36,38,40,42-45,48,51], and 1 analytical cross-sectional study [34]. The participant samples predominantly consisted of undergraduate nursing and/or medical students at various stages of their training. The objectives of the studies commonly focused on evaluating the use of simulation-based training on competencies essential for patient safety, such as interprofessional communication, teamwork, and clinical reasoning. The studies that included only nursing students were 14 [32-34,36-46] and the remaining 6 were studies with a mixture of nursing and medical students [35,47-51], although one of them only included 1 medical student [50].

The total number of participants included was 2494. The study with the least participation grouped 30 students [41] and the one with the highest participation had 231 [42]. Most participants were women (more than 60%); in one of the studies, all participants belonged to a women’s nursing school [41].

The studies were carried out from 2015 [34] to 2023 [35]; 5 of them did not report the age of the participants, and 4 did not report the academic year in which the participants were enrolled, their sex, or the year the study was conducted.

Measurement tools varied across studies. Only 4 studies used ad hoc questionnaires [34,36,38,44]. The remaining 16 studies [32,33,35,37,39-43,45-51] used validated instruments, some with contextual adaptations (eg, cultural and language modifications and scenario-specific items).

### Characteristics of Interventions

The simulation-based training interventions detailed in the included studies were heterogeneous, as detailed in Table 4. Interventions ranged from single-session workshops (eg, 3.5 h) to multisession programs integrated into the curriculum.

The modalities of simulation used included high-fidelity simulators (HFS; n=9) [32,34,39-41,43,45,47,48], SPs (n=8) [35-39,48,49,51], virtual reality (VR; n=4) [44-46,49], role play (n=2) [38,50], and board simulations (n=1) [33]. Several studies combined HFS with SP or VR, so the categories are not mutually exclusive. The transfer of communication using a structured methodology between the team uses different strategies, such as SBAR (Situation, Background, Assessment, Recommendation) and SBAR-R [32,34,37,47,50] handover tools and KidSIM-TPS [50] teamwork frameworks, while other less commonly used techniques include the Kalamazoo communication checklist [46]. Structured communication with the patient is guided by the SEGUE (Set the stage, Elicit information, Give information, Understand the patient’s perspective, End the encounter) framework [36]. These structured tools were detailed in the instruments and notes of Table 3.

**Table 4.** Characteristics of interventions.

Author (year), country	Modalities and type of simulation	Security aspects covered	Direction of effect	Type of intervention in the study	Case
Breen et al [47] (2019), Ireland	HFS^a^-HF^b^	Communication between professionals	Favors competency-based simulation	All groups 15 min training on the ISBAR^c^ tool. Group (E^d^): HF room. Group (E+S^e^) work in mixed discipline pairs: telephone calls on 4 standardized cases (3.5 h). Group (E+PBP^f^): same training as group E+S, reached competencies	4 standardized clinical cases of acute patient deterioration
Son and Kim [36] (2019), Korea	SP^g^-HF	Communication with the patient	Favors simulation	CG^h^: Prebriefing + pretest (50 min), simulation 60 min, debriefing+posttest (50 min) afterward, and SEGUE^i^-based communication (30 min). IG^j^: SEGUE communication before	Standardized pediatric scenario, with mothers of 5-year-old children admitted to the hospital for acute gastroenteritis with fever
Jeong and Kim [37] (2020), Korea	SP-HF	Falls and communication	Favors simulation	IG: 3 educational sessions on falls communication and SBAR^k^ method (theory, practice, and discussion). CG: training according to guidelines on patient care and transfer of information	Standardized patient falling out of bed
Liaw et al [49] (2020), China	VR^l^ (simulated environment with avatar use) and live simulation (SP)-HF	Interprofessional communication and teamwork	Noninferior (VR vs live)	All groups received 3 h of team training on nurse-physician communication. IG: virtual environment and avatar, CG: SP. Each scenario lasted approximately 15‐20 min and was followed by a 30-min facilitator briefing	Sepsis and septic shock scenario
Sanko and McKay [33] (2020), United States	Board simulation (board game) to teach and develop systematic thinking-LF^m^	Notification of incidents and adverse effects. System failures	Favors simulation	CG: Simulation scenarios. IG: 2017 course included a tabletop simulation“ Friday Night in the ER.”	Emergency care
Lee and Kim [34] (2020), Korea	HFS (IAM^n^)-HF	Interprofessional communication	Positive association	Before the scenario training on SBAR-R^o^ communication and election of the leader of each team. Development of the scenario in 2 times: before a call to a fictitious doctor and performance of the task in a team after receiving instructions by phone	Acute myocardial infarction emergency care
Wai et al [48] (2021), Korea	HFS-HF	Teamwork	Both improved	IG: combined classroom+simulation. CG 1: online training on team-based learning, individual test and later in group, with those who would solve the simulation. CG 2: clinical case simulation. All groups are training on patient safety, human factors, and communication	Predetermined critical case scenarios: chest pain and weakness in MMII^p^
Musharyanti et al [38] (2021), Indonesia	Role play and simulated patients (actors)-LF	Medication administration	Favors simulation	IG: 18-h training sessions over 5 wk, overview and training activity with a 4C/ID^q^ approach including presentation of real cases, small group discussion, reflection and simulation of oral and intramuscular drug administration. CG: 2 wk, overview, 2 lecture sessions and video playback, and posttesting	Oral and intramuscular drug administration
Du et al [39](2021), China	SP in high fidelity room-HF	PUP^r^	Favors simulation	CG: pressure ulcer training in the conventional classroom 90 min, IG: training through simulated clinical scenarios	Three clinical scenarios from admission, hospitalization and deterioration of the disease
Lee and Lim [32] (2021), Korea	HFS (respiratory)-HF	Communication in transfer	Favors simulation	Students were divided into groups of 3 to 4 for 120-min sessions consisting of 50 min of theoretical training and 70 min of simulation-based training	2 patients with respiratory problems with high-fidelity simulator
Craig et al [40] (2021), United States	HFS with computer package, electronic medical records (identification wristbands, carts, barcodes, and computerized records)	Medication administration	Favors simulation	4 wk of simulation. IG: 1. low-fidelity simulation on medication administration. 2. high-fidelity simulation focused on safe medication administration. 3. clinical rotation. 4. high-fidelity simulation+debriefing. CG: 1. standard training. 2 clinical rotation and 3‐4 same as IG	Scenario for administration of oral and subcutaneous medication (insulin aspart)
Raurell-Torreda et al [50] (2021), Spain	Role-playing SBAR. - LF	Interprofessional communication	Favors simulation	IG: divided into subgroups of 20 students for 1-h role play session, learning objectives focused on basic professional health care skills, teamwork, use of SBAR worksheet and role distribution in respiratory tract management, nursing procedures and techniques, and use of documentation. Patient assessment and intervention in 3 nursing roles: procedures, assessment, and follow-up	Patient in shock in an emergency department setting (based on a clinical case from the National League for Nursing) to assess teamwork and non-technical skills
Park and Kim [41] (2021), Korea	HFS (mannequin)-HF	Systemic and organizational factors. Diagnostic errors	Favors simulation	IG: The PDS-IB^s^ and CG: simple PDS. The scenario theme for the simulations in both groups was patient deterioration. The simulations for both groups consisted of 1.5 h	Patient with chronic obstructive pulmonary disease was transferred from the emergency room to the inpatient ward (worsening)
Chen et al [51] (2022), Taiwan	SP-HF	Teamwork	Comparable gains	In group 1 (received IPE^t^ training, followed by SPE^u^) and group 2 (received SPE training followed by IPE training). Simulation training was structured for 4 wk (3 h/wk) that incorporated a 2-wk IPE program during which medical and nursing students were trained together	Critically ill patients with AHA^v^ guidelines for cardiopulmonary resuscitation and emergency cardiac care
Pol-Castañeda et al [42] (2022), Spain	SP (clinical case)-HF	Medications	Favors simulation	Briefing, simulation scenarios were conducted in 24 groups of 6 to 8 students, each playing a different role	3 scenarios: hypocalcemia due to gastrointestinal disease in the emergency department, respiratory infection, paracentesis
Goldsworthy et al [44] (2022), Canada, England, Scotland, and Australia	VR-HF	Patient deterioration	Favors virtual simulation	The treatment group completed 6 VR of medical surgical nursing case studies over 3 wk (2/wk). Two VR were completed each week that they could repeat	Acute deterioration care: angina and cardiac arrest; anaphylaxis; acute asthma exacerbation; COPD^w^ and pneumothorax, pulmonary embolism; and blood transfusion reaction.
Li et al [43] (2023), China	HFS (standardized patient+mannequin)-HF	Patient safety, medication errors, and adverse effects	Favors combined training	All: online course-training adverse effects, types, effects, and communication and teamwork. IG: 2 simulation cases in addition to training. CG: online training only	Scenarios of care in respiratory infection (medication) and hemiplegia (basic care)
Haerling et al [45] (2023), United States	HFS mannequin and VR displays-HF	Safety risks. Interprofessional and patient communication Medication Administration	Manikin superior and equivalent	Clinical experience: 4 h of traditional clinical experience Mannequin simulation: simulation activities with mannequins in 2 scenarios VR was similar to that of the mannequin-based simulations. The groups varied according to the type of experiential learning activity they completed first	Postoperative discharge care and postoperative emergency room readmission
Chou et al [46] (2024), Taiwan	VR-HF	Communication	Favors VR simulation	IG received a VR training in nurse-patient communication skills 2 wk prior to practice. The program was delivered in 4 sessions for 30 min each time for 2 wk. CG received the 30-min nurse-patient communication teaching video that could be downloaded and viewed	Simulated hospital ward scenarios with 4 learning tasks: self-presentation, establishing a nurse-patient relationship, interaction, and medical history collection
Heier et al [35] (2024), Germany	SP-HF	Notification of medication errors	Favors simulation	Interprofessional communication skills training on acute care medical errors (IG) with a cohort that did not receive interprofessional training (CG)	3 scenarios reported in a critical incident reporting system focused on medication errors caused by a chain of errors. Chemotherapy, wrong antibiotic, and chemotherapy preparation with errors

a HFS: high-fidelity simulator.

b HF: high-fidelity.

c ISBAR: Identity-Situation-Background-Assessment-Recommendation.

d E: e-learning.

e E+S: e-learning plus standard simulation group.

f E+PBP: e-learning plus competency-based progression simulation group.

g SP: standardized patient.

h CG: control group.

i SEGUE: Set the stage, Elicit information, Give information, Understand patient perspective, End the encounter.

j IG: intervention group.

k SBAR: Situation, Background, Assessment, Recommendation.

l VR: virtual reality.

m LF: low fidelity.

n IAM: High Fidelity Simulator for Acute Myocardial Infarction.

o SBAR-R: SBAR with Readback and Response.

p MMII: Lower Limbs.

q 4C/ID: Four Components Instructional Design.

r PUP: pressure ulcer prevention.

s PDS-IB: Patient Deterioration Simulation with Inattentional Blindness.

t IPE: Interprofessional Education.

u SPE: Single Profession Education.

v AHA: American Heart Association.

w COPD: chronic obstructive pulmonary disease.

### Methodological Quality Results

The overall methodological quality of the studies was moderate to high. Using the JBI Critical Appraisal Tools, RCTs generally scored between 9 and 13 out of 13 possible criteria, quasi-experimental studies between 7 and 9 out of 9, and cross-sectional studies met 6 to 8 out of 8 criteria, assessed according to JBI methodological standards for evidence synthesis [29-31].

Common strengths included clear description of inclusion criteria, valid measurement of outcomes, and appropriate statistical analyses. The most frequent limitations were related to lack of blinding of participants or assessors, incomplete follow-up, and limited control of confounding factors.

A summary of the appraisal results is presented in Table 5, while full details of the assessment can be found in Tables S2, S3, and S4 in Multimedia Appendices 3-5.

**Table 5.** Summary of methodological quality results.

Study design	Number of studies	JBI^a^ tool used	Mean criteria met (%)	Common strengths	Common limitations
Randomized controlled trials	8	JBI RCT^b^ Checklist (13 items)	85‐100 (9, 13/13)	Clear objectives, valid measurement tools, and appropriate statistical analysis	Lack of blinding (3), small samples
Quasi-experimental studies	11	JBI Quasi-Experimental Checklist (9 items)	78‐100 (7, 9/9)	Clear cause-effect design and reliable outcome measures	Absence of control group in some cases (3), incomplete follow-up (2)
Cross-sectional studies	1	JBI Analytical Cross-Sectional Checklist (8 items)	75 (6/8)	Clear inclusion criteria, valid exposure, and outcome measures	Confounding not always controlled

a JBI: Joanna Briggs Institute.

b RCT: randomized controlled trial.

### Topics

The topics they discussed were communication, both interprofessional [32,34,46,47,49,50] as communication with the patient [36], teamwork [48,51] aspects related to medication, such as its administration [38,40,42] and notification of errors or adverse effects [33,35,43], systemic and organizational factors [41], falls [37], pressure ulcer prevention [39], patient deterioration [44], and various security aspects a mix of the above [45].

Mixed groups, with nursing and medical students, address topics such as interprofessional communication and teamwork [48-51]; only one of them addresses the issue of medication [35].

Most simulations aimed at developing communication and teamwork skills used high-fidelity modalities, including SP and mannequins [32,34,36,47,48,51] and VR environments [46,49]. One study used role-playing as the primary simulation modality [50].

To develop skills in aspects of medication administration, HFS has been used [40], role play [38], and simulated patients [38,42]. In the case of notification of medication errors and adverse effects, the simulation scenarios have used SPs and mannequins [35,43] and a board game [33]. The rest of the safety aspects discussed have used simulated patients or mannequins, except the study on patient deterioration, which has used VR technology [44].

High-fidelity simulation through HFSs, VR, and SPs predominated across studies. However, low-fidelity approaches, such as role-playing, were also used, either alone or combined with other modalities, particularly in interprofessional communication [50], medication administration [38], and incident or system failure reporting [33].

### Safety Outcomes

In general, almost all studies link training through simulation scenarios with an improvement in safety-related skills and knowledge [32-47,50]. One study directly compared live (face-to-face) simulation with immersive VR simulation in scenario-based training [49]. The comparator was therefore 2 different simulation modalities rather than simulation versus traditional teaching. No statistically significant differences were found between live and VR simulation in the development of teamwork and communication skills. These findings suggest that VR-based simulation may represent a comparable alternative to live simulation for fostering these competencies.

Simulation training complemented by other techniques (lectures, presentations, debates, and demonstrations) achieves better results than simulation training alone for improving patient safety competency among nursing students [43]. However, the effects of a blended classroom plus clinical simulation versus clinical simulation alone on teamwork attitudes did not further improve teamwork attitudes, perceptions, and performance in medical and nursing students compared with clinical simulation alone [48]. A quasi-experimental study comparing traditional clinical practice, manikin-based high-fidelity simulation, and screen-based virtual simulation (n=193) found no significant differences in cognitive outcomes between groups [45]. However, students in the manikin-based simulation group achieved significantly higher scores than those in the traditional clinical group in several competency domains measured by the Creighton Competency Evaluation Instrument, including communication (effect size=0.52; *P=.*04), although no significant differences were observed in the Patient Safety domain. These findings suggest that the type of simulation experience may influence specific clinical competency outcomes [45].

Only 2 studies followed up over time after training [46,49]. In one study, the results were better than before after 1 week of practice [46]. A RCT compared immersive VR simulation with conventional live simulation in 120 medical and nursing students. Both modalities significantly improved teamwork attitudes and interprofessional socialization immediately postintervention. At 2-month follow-up, only the VR group maintained a significant improvement in Interprofessional Socialization and Valuing Scale scores (*P*=.047), although no statistically significant differences were found between groups at any time point, suggesting comparable effectiveness [49].

Two studies evaluated competencies using Objective Structured Clinical Examination–based assessments. In one study, 47 second-year nursing students receiving simulation-based training achieved significantly higher scores in pressure ulcer risk assessment than those receiving standard instruction (mean 29.04, SD 6.00 vs mean 12.38, SD 4.15; *P*<.001) across 3 simulated scenarios using SPs [39]. In another randomized educational trial including 90 nursing and medical students, competency-based simulation combined with e-learning led to higher communication competence (60%) compared with e-learning alone (6.9%) and standard simulation (13%), using a clinical structured communication tool [47].

## Discussion

### Principal Findings

This systematic review aimed to evaluate the effect of simulation-based education on patient safety outcomes in undergraduate nursing students, to identify the patient safety domains addressed through simulation, and to describe the characteristics of simulation-based educational interventions. Overall, the findings suggest that simulation-based education is associated with improvements in several competencies related to patient safety, particularly communication, teamwork, medication safety, and the recognition and reporting of clinical incidents. High-fidelity simulation predominated among the included interventions, with 16 studies using this modality [32,34-43,45,47,48,50,51]. In addition, the incorporation of VR [35,37,40] expands the range of clinical scenarios that can be recreated in educational environments and may facilitate the development of knowledge and skills related to patient safety [43].

Among the patient safety domains addressed, communication and teamwork were the most frequently studied. Several studies reported improvements in these competencies following simulation-based training among nursing and medical students [38-42]. Simulation scenarios provide opportunities for students to practice interprofessional collaboration and structured information transfer in controlled learning environments. Structured communication tools, such as SBAR and its adaptations, were frequently incorporated into simulation scenarios and were associated with improved team coordination and information transfer [32,34,37,46,47,50]. Similarly, structured communication strategies have been shown to improve communication between students and patients [36]. Communication-focused simulations often involved interprofessional scenarios in which students practiced structured communication during clinical deterioration or emergencies, strengthening collaborative decision-making between professionals [47,49,50].

Medication safety was another frequently addressed domain. Three studies evaluated students’ ability to safely administer medications, identify potential errors, and follow appropriate safety procedures [38,40,42]. Simulation provides a controlled environment in which students can practice medication administration while recognizing potential safety risks without endangering real patients. In addition, simulation-based education was associated with improvements in the reporting of adverse events and medication-related incidents [40,42]. Other 3 studies also explored the development of systems thinking and incident reporting competencies through simulation scenarios focused on adverse event reporting or clinical error disclosure [33,35,43]. These activities encouraged students to recognize systemic factors contributing to patient safety incidents and to adopt a nonpunitive perspective toward error management.

This review also highlights the diversity of simulation modalities used to address patient safety competencies. SPs were commonly used to train communication with patients [36], reporting incidents such as falls or medication errors [35,37], pressure ulcer prevention [39], teamwork and interprofessional communication [49,51], and medication administration [38,42]. High-fidelity simulation using mannequins was the most frequently used modality [32,34,39-41,43,45,47,48]. VR simulations were also used, particularly in scenarios focused on communication and teamwork [35,46,49].

Although high-fidelity simulation was widely used, lower-technology simulation approaches also demonstrated positive learning outcomes. Role-playing, tabletop simulations, and screen-based virtual simulations improved competencies related to communication, systems thinking, and incident reporting [33,38,50]. These findings suggest that simulation-based education can be implemented across a variety of educational settings, including institutions with limited technological resources. In addition to simulation modality, the instructional design of the intervention influenced outcomes. One study reported greater improvements when simulation was integrated with complementary educational strategies such as lectures, structured feedback, or online modules [43].

### Comparison With Previous Literature

The findings of this review are consistent with previous literature highlighting the educational value of simulation in health professions education [5252]. Improvements in communication and teamwork competencies observed in the included studies align with recent meta-analyses emphasizing the importance of interprofessional education for improving role understanding and collaborative practice among health care students [53,54]. Improvements in these competencies are particularly relevant to patient safety, as failures in communication and teamwork are frequently associated with preventable adverse events in clinical practice. Interprofessional simulation experiences have been shown to strengthen role clarity and collaborative practice, thereby promoting safety-oriented behaviors among future health professionals [54].

Similarly, the improvements observed in medication safety practices are consistent with previous reviews suggesting that simulation-based education can be an effective method for improving patient safety competencies [24]. Evidence from systematic reviews also indicates that the use of SPs may enhance communication skills, learning outcomes, and problem-solving abilities in health professions education [23]. Previous reviews have also reported improvements in knowledge, confidence, and clinical skills following simulation-based learning [21,22], although many of these studies did not specifically focus on patient safety outcomes. Compared with earlier literature, the present review provides a more focused synthesis of evidence regarding the use of simulation-based education to address patient safety competencies in undergraduate nursing students.

The findings of this review also suggest that the use of different simulation methods and varying levels of fidelity, as observed across the included studies, can be similarly effective for developing competencies related to patient safety. These results are consistent with previous literature, indicating that the effectiveness of simulation-based education does not depend solely on the level of technological fidelity. Studies comparing high-fidelity simulation with alternative teaching strategies, such as written case studies, have not demonstrated clear advantages of high-fidelity simulation alone for improving critical thinking skills in nursing students [55].

### Sustainability of Training Effects

Despite the positive outcomes reported in most studies, the long-term sustainability of simulation-based training effects remains unclear. Only 2 studies included follow-up assessments after the intervention, with relatively short follow-up periods of 1 week and 2 months [46,49]. Consequently, it is difficult to determine whether the improvements observed are maintained over time or translate into sustained behavioral changes in clinical practice. This limitation is particularly relevant because previous research on medication safety suggests that safety-related competencies may decline if they are not reinforced through continued practice and training [56].

### Practical Implications for Nursing Education

The findings of this systematic review have several important practical implications for undergraduate nursing education. Overall, the evidence indicates that simulation-based education is an effective strategy for developing key patient safety competencies, particularly in communication, teamwork, medication safety, and error recognition [32-47].

The results support the systematic integration of simulation-based learning into nursing curricula, rather than its use as an isolated or supplementary teaching activity. Simulation-based interventions embedded within educational programs improved students’ patient safety competencies [40,42,43,45,46]. Simulation scenarios focused on real-world patient safety challenges enable students to apply theoretical knowledge in a safe and controlled environment [39,41,44].

The impact of structured communication tools, such as SBAR, ISBAR (Identification, Situation, Background, Assessment, Recommendation), and SEGUE, suggests that these frameworks should be explicitly incorporated into simulation-based training [32,34,36,37,47]. The use of these tools during simulation may reduce communication-related errors and enhance patient safety.

In terms of the characteristics of the simulation, the included studies used various methods, including manikin simulation, SPs, virtual simulation, role-playing, and tabletop simulation. The level of fidelity within these modalities varied between low, medium, and high, depending on the technological complexity and degree of clinical realism described by the authors. While experiences with a higher level of technological fidelity were common, several studies demonstrated that modalities with lower technological requirements, such as role-playing or tabletop simulation [33,38,50], as well as basic virtual environments [44-46,49], could also be effective in improving patient safety knowledge and skills. These findings suggest that meaningful educational outcomes can be achieved with modalities that have lower technological requirements, which is particularly relevant for institutions with limited resources.

### Current Gaps in the Literature

Even with mounting evidence backing simulation-based training in nursing, this review identified several important gaps in existing literature. One of the most significant limitations is the lack of long-term follow-up. Only 2 of the studies included assessed outcomes beyond the immediate postintervention period, with follow-up periods of 1 week and 2 months, respectively [46,49].

Another gap relates to study design and methodological rigor. Although RCTs were included, 9 of studies used quasi-experimental designs with convenience sampling and relatively small sample sizes [24,25,29-31,33-36]. In addition, blinding of participants and assessors was rarely reported. Also, studies used a wide range of instruments to assess patient safety competencies, including self-developed questionnaires and modified scales [25,27,29,33,35].

Geographically, most studies were conducted in high-income countries [22-42], with limited representation from low- and middle-income settings, indicating a lack of global diversity in the evidence base.

### Recommendations for Future Research

Future research should address the limitations identified in this review. Longitudinal studies are needed to assess retention and maintenance of competencies. The use of standardized tools and methodologically sound, multicenter clinical trials would improve external validity and avoid bias. On the other hand, studies comparing the efficiency and cost-effectiveness of different simulation modalities would help guide curricula and resource allocation. It would be necessary to include other educational contexts in low- and middle-income countries in order to apply the results and guide nursing training plans. The limited reporting of facilitator preparation across studies highlights the need for future research to examine the role of educator training in simulation-based patient safety education.

### Limitations

It is important to consider the limitations of this study when interpreting the results. First, there is a considerable degree of heterogeneity between the studies, not only in terms of the type of intervention and duration, but also in terms of the training of the participants, experience, and origin. Additionally, the variability of measurement instruments, convenience samples, and the use of self-reported data may introduce bias. Furthermore, the restriction of the search to studies published in English, Spanish, and Portuguese may have resulted in the exclusion of some relevant studies. The criteria used to establish the cutoff point in the methodological quality assessment may have influenced the final selection of evidence included.

The search strategy was limited to the terms “patient safety” and “safety,” which may have led to the exclusion of relevant studies addressing patient safety outcomes. Although additional manual screening of references was performed to identify potentially relevant studies, this restriction may have reduced the sensitivity of the search.

Although a systematic approach to study selection ensures the quality of the evidence, it may result in the omission of relevant research that, although less rigorous, could offer valuable insights.

### Conclusions

Despite considerable discrepancies between individual studies, simulation is an important tool that empowers nursing students to identify, mitigate, or eliminate potential risks to patient safety. The use of simulation methodology facilitates the acquisition of essential competencies, including communication skills, teamwork, medication administration, and error detection and adverse effect recognition. These skills are crucial for ensuring patient safety upon entering the professional workforce. The use of high-fidelity simulation enables the recreation of clinical scenarios, facilitating the integration of theoretical and practical training, and thus the development of skills and a more comprehensive integration of knowledge.

Simulation-based education improves nursing students’ competence in key safety domains (communication, teamwork, medication safety, and error recognition), thus contributing to improved patient safety outcomes.

## Supplementary material

10.2196/87898 Multimedia Appendix 1 Characteristics of the studies.

10.2196/87898 Multimedia Appendix 2 Characteristics of the interventions.

10.2196/87898 Multimedia Appendix 3 Quality assessment 1.

10.2196/87898 Multimedia Appendix 4 Quality assessment 2.

10.2196/87898 Multimedia Appendix 5 Quality assessment 3.

10.2196/87898 Checklist 1 PRISMA checklist.

## Abbreviations

HFS: high-fidelity simulator
ISBAR: Identification, Situation, Background, Assessment, Recommendation
JBI: Joanna Briggs Institute
MeSH: Medical Subject Headings
PIO: Patient, Intervention, Outcome
PRISMA: Preferred Reporting Items for Systematic Reviews and Meta-Analyses
RCT: randomized controlled trial
SBAR: Situation, Background, Assessment, Recommendation
SEGUE: Set the stage, Elicit information, Give information, Understand the patient’s perspective, End the encounter
SP: standardized patient
VR: virtual reality
WHO: World Health Organization
