# Supplementary material for: Simulation-Based Training for Nursing Students to Improve Patient Safety: Systematic Review
Source: JMIR Nurs. 2026 May 26;9:e87898. doi: 10.2196/87898 (PMC13205464; doi:10.2196/87898)
Supplement: Multimedia Appendix 1 [file nursing-v9-e87898-s001.pdf]

Table S1. Characteristics of Included Studies

Author (s)Year, Country	Participants	Type of study/assignment	Objectives	Data collection scales/instruments	Results and Conclusions	Limitations
Breen et al.2019 Ireland .[47]	Total 90: 45 nursing students3rd course –45 last year medical students. Year: 2016.	ECA 3 groups: e-learning (E), E +standard simulation (E+S), competency-based progression simulation and e-learning (E+PBP). Randomized	To determine the effectiveness of a competency-based training (PBP) for clinical communication.	National Early Warning Score (NEWS) based on the ISBAR tool.	Communication competence was achieved by 2/29 (7%) in e-learning, 3/23 (13%) in e-learning plus simulation, and 15/25 (60%) in competency-based progression. Competency-based training was significantly more effective (p<0.001)	Single centre; undergraduate sample; short training duration.
Son & Kim, 2019 Korea [36]	98 Nursing students. CG:41, IG: 57 Course 3rd. Year: 2017 Age: 22-23 Gender: Women	Cuasi-experimental. Pretest-post test design of non-equivalent control group. Convenience sampling	Effectiveness of communication education based on SEGUE between students and patient.	Communication competence and effectiveness measured by a self-report questionnaire and by teacher and standardized patient ratings of the students	Intervention students showed significantly greater improvements in communication competence and efficacy than controls (all p<0.05).	Single institution; small control group.

Jeong & Kim, 2020 Korea [37]	54 nursing students. IG: 26, CG: 28 Grade: 3rd Year: 2018 Age: 20-40 average 23 Gender: Women, only 1 man	Clinical trial (pre-post). Simple blind	Develop a fall simulation program using the SBAR communication technique	Knowledge of falls and attitude towards falls scale, post-fall evaluation protocol (AHRQ). GICC-15 and Dunsford adapted structured communication	SBAR-based simulation significantly improved structured fall-related communication compared with controls.	No long-term follow-up; single-blind design.
Liaw et al. 2020 China [49]	120 medical and nursing students. IG: 60, CG: 60 Grade: 3rd-4th year. Year: 2018 Age: mean 22.17 years (SD 2.07). Gender: Women (81, 67.5%)	RCT (pretest-post test) Randomized	To evaluate a team training program using VR versus conventional live simulations on the performance of communication skills and teamwork	Attitudes Toward Interprofessional Health Care Team (ATHCT) and Interprofessional Socialization and Valuing Scale (ISVS). Baseline, post-test and 2 months after	Both groups improved teamwork attitudes; VR training was non-inferior with higher follow-up ISVS	Self-reported outcomes; single-centre design.
Sanko & McKay ,2020 USA [33]	231 Nursing students from 2016 (CG: 68),	Clinical trial (2 cohorts years different intervention and	To assess whether exposure to simulation scenarios to	Simulated Adverse Event Reporting System (AERS). System	Systems thinking scores increased significantly after simulation	Single-site sample; simulated reporti

	2017 (IG: 85) and 2018 (CG: 78) patient safety courses.	control groups). Convenience sampling	enhance systematic thinking influenced adverse event reporting and the type	Thinking Scale (STS).	and adverse event reporting improved (p<0.001).	ng system.
Lee & Kim, 2020 Korea [34]	194 nursing students. 47 teams (4 and 3 components) Course: higher level. Year: 2015-2016	Prospective observational Convenience sampling	To examine the relationships between nursing students' team task performance and SBAR-R communication.	Measurement of team task performance and communication using developed checklists based on SBAR-R.	Higher SBAR-R scores were significantly associated with better team task performance (p<0.05).	Single university; limited scope.
Wai et al. 2021 Korea [48]	46 students: 19 medical, 27 nursing. Interprofessional teams. Grade: Final year medicine (5th), 3rd-4th year nursing. Gender: 63% female students.	Mixed methods Convenience sampling.	To compare the effectiveness of combined classroom plus clinical simulation versus clinical simulation alone on attitudes perceptions and teamwork performance.	HFAS Survey. Teamwork Performance using TBL- SAI Focus group interview	Both groups improved teamwork attitudes (p<0.05) with no added benefit from classroom teaching	Small sample; limited power.

Musharyanti et al. 2021 Indonesia [38]	95 nursing students IG:55. CG: 40.	Quasi-experimental. Non-equivalent randomized control group.	To compare drug safety knowledge and skills after safety training with the 4C/ID teaching method.	Multiple choice questionnaires (MCQ) and two checklists developed ad hoc on patient safety and medication administration.	Intervention students achieved significantly higher safety knowledge and skills than controls ($p<0.001$).	No baseline testing
Du et al. (2021) China [39]	47 nursing students.CG:21, IG:26 Grade: 2nd Year 2019. Age 17-27 Gender: 44 women -3 men	Controlled trial. Simple blind.	To assess the risk of pressure ulcer development in three different scenarios	OSCE adapted to different scenarios Pressure Ulcer Knowledge Assessment Tool (PUKAT 2.0)	OSCE-based simulation significantly improved assessment performance ($p < 0.001$).	Small sample. Single centre
Lee & Lim, 2021 Korea [32]	30 nursing students. Grade: Final year. Year: 2018. Average age 22.17 Gender: women	Quasi-experimental(pre-post). Convenience sampling	Develop, implement and verify the effectiveness of a simulation-based handover education program.	Communication tool adapted from SBAR. Knowledge questionnaire developed by authors. Self efficiency (adapted questionnaire).PASS-BAR	Simulation significantly improved handover knowledge, self-efficacy and performance ($p \le 0.001$).	No control group; all-female sample.

Craig et al. 2021 USA [40]	83 nursing students. CG:35. IG:45 Grade: 3rd.	Quasi-experimental. Convenience sampling	To examine the effects of an educational strategy using a simulation program on medication management.	MSKA and MSCEC	Simulation significantly improved medication administration skills ($p<0.001$).	Single-site study
Raurell-Torreda et al. 2021 Spain [50]	93 nursing students. IG: 48, CG:45 Course: 3rd, 1 medical student in 5th year. Age: CG: 22.3 ± 5.2- IG: 23.3 ± 6.8 Gender: 78.5% women	Clinical Trial Randomized	Evaluate the impact of SBAR training on interprofessional teamwork skills (role-related and communication) and non-technical skills.	KidSIM – TPS and CSET (non-technical skills)	Intervention students showed significant improvements in teamwork behaviors ($p\leq0.004$).	Partial implementation.
Park & Kim, 2021 Korea [41]	91 nursing students. GI: 47, GC: 44. Final year of nursing Academic year 2018/19. Age: IG 22.59	Clinical Trial Randomized	Analyze the impact of simulated patient deterioration on situational awareness and patient safety competence-attitude.	SAGAT modified; PSCSE modified.	Simulation significantly improved situational awareness and safety attitudes ($p<0.001$).	Single university.

	±1.23, CG 22.86±1.39 Gender: +77% women					
Chen et al. 2022 Taiwan [51]	54 students: 18 medical students and 36 nursing students Grade: 4th year nursing, 5th year medicine Year: 2019 Gender: 70% Women	Mixed methods Randomized	Determine the importance of interprofessional training on competence, teamwork attitudes and safety.	MTP, TBP, TA and PSA	Both groups achieved comparable competence gains; qualitative data supported learning benefits	Small sample
Pol-Castañeda et al. 2022 Spain [42]	179 nursing students. Grade: 2nd Academic year 2018/19 Age: 60% between 18-25 Gender: 89% women,	Mixed methods Convenience sampling	To assess the acquisition of skills in safe medication administration by nursing students	Questionnaire. adapted from the. MASAT	Simulation improved most medication skills except documentation.	Assessment variability.
Goldsworthy et al. 2022	88 nursing students five	Quasi experimental Conveni	Explore the impact of a virtual simulation	10-item Clinical Self-Efficacy	Virtual simulation significantly improved	Non-randomized design.

Canada England Scotland and Australia [44]	diverse university sites in four countries Grade: 3rd-4th Pandemic year 2020?	ence sampling	on to recognize and respond to a rapidly deteriorating patient.	Survey designed 20-item multi-choice test on evidence-based practice.	knowledge scores (p=0.001).	
Li et al. 2023 China [43]	205 nursing students.IG:103, CG: 102. Grade: 2nd Academic year2020/21. Mean age CG: 19.65± 0.75 IG:19.78 ±0.77 Gender: women 87%-89%.	Quasi-experimental (2-tailed) Convenience sampling	Exploring the effects of an online course (SPOC) combined with simulation-based training in a patient safety education program.	PSCSE	Patient safety competence scores were significantly higher in intervention students (p<0.001).	Single-centre study.
Haerling et al. 2023 USA [45]	193 nursing students: Group Clinical experience: 51, Group Mannequins:44, Group	Quasi experimental Convenience sampling	Compare differences in learning and practice in patient care as a function of the learner's training experience.	Competency by CCEI and LCJR. Clinical Learning CLECS 2.0	Manikin-based simulation produced equal or superior outcomes compared with other modalities (p<0.05).	Regional sample.

	VR: 57. Academic year 2021/22.Age: 19-53(median =21). Gender: 81.6% female					
Chou et al. 2024 Taiwan [46]	84 nursing students IG: 42 and CG: 42. Grade: 2nd Year: 2022. Mean age20.3 years (SD = 0.46). Gender: 80% women	Clinical Trial Randomized	To examine the effectiveness of a VR communication simulation in the acquisition of communication skills in the fundamentals of nursing practice.	Kalamazoo Consensus Statement Essential Elements Communication Checklist, "Communication Self-Assessment Scale" modified, Development and Testing of a perceived Stress Scale for Nursing Students in Clinical Practice and a learning satisfaction	Intervention students showed significantly higher communication skills and lower stress (p<0.001).	Short intervention; self-report bias.
Heier et al. 2024 Germany [35]	221 Students, 154 medical and 67 nursing students (IG: 66 medical, 28 nursing / CG:	Mixed methods Convenience sampling	Develop joint communication skills training for nursing and medical students in professional error communication.	Adaptation of G- IPAS "Teamwork,Roles and Responsibilities", "Patient-centeredness" and a self-developed interprofessional error communication scale.	Interprofessional communication and teamwork	Non-randomized design.

	88 medical, 39nursing) Course: 3rd year medical and 1st - 2nd year nursing students. Year: October 2021-March 2023.Mean age24 (SD: 3.9). Gender: 51.13% women			attitudes improved significantly ($p \le 0.012$).	

Note: RCT: Randomized Controlled Trial; IG: intervention group, GC: control group E: e-learning; 4C/ID: Four-Component Instructional Design; AERS: Adverse Event Reporting System; AHRQ: Agency for Healthcare Research and Quality; ATHCT: Attitudes Toward Interprofessional Health Care Team; CCEI: Creighton Competency Evaluation Instrument; CG: Control Group; CI: Confidence Interval; CLECS: Clinical Learning Environment Comparison Survey; CSET: Clinical Simulation Evaluation Tool; E: E-learning group; E+PBP: E-learning plus competency-based Progression simulation group; E+S: E-learning plus Standard simulation group; G-IPAS: German Interprofessional Attitudes Scale; GICC-15: General Interpersonal Communication Competency (15 items); HFAS: Human Factors Attitude Survey; IG: Intervention Group; ISBAR: Identification, Situation, Background, Assessment, Recommendation; ISVS: Interprofessional Socialization and Valuing Scale; KidSIM-TPS: KidSIM -Program Team Performance Scale; LCJR: Lasater Clinical Judgment Rubric; M: Mean; MASAT: Medication Administration Safety Assessment Tool; MCQ: Multiple Choice Questionnaire; MD: Mean Difference; MSCEC: Medication Safety Critical Elements Checklist; MSKA: Medication Safety Knowledge Assessment; MTP: Medical Task Performance; NEWS: National Early Warning Score; OSCE: Objective Structured Clinical Examination; PASS-BAR: Patient Safety Screen-Based Assessment Record (handover tool); PBP: Progression-Based Performance / Competency-Based Progression; PSA: Patient Safety Attitude; PSCSE: Patient Safety Competency Self-Evaluation; PUKAT: Pressure Ulcer Knowledge Assessment Tool; RCT: Randomized Controlled Trial; SAGAT: Situational Awareness Global Assessment Technique; SBAR:

Situation, Background, Assessment, Recommendation; SBAR-R: Situation, Background, Assessment, Recommendation, Read-back; SD: Standard Deviation; SEGUE: Setting the stage, Eliciting information, Giving information, Understanding the patient's perspective, Ending the encounter; SPOC: Small Private Online Course; STS: Systems Thinking Scale; TA: Teamwork Attitude; TBL-SAI: Team-Based Learning Student Assessment Instrument; TBP: Team Behaviour Performance; VRCS: Virtual Reality Communication Simulation
