# Supplementary material for: Simulation-Based Training for Nursing Students to Improve Patient Safety: Systematic Review
Source: JMIR Nurs. 2026 May 26;9:e87898. doi: 10.2196/87898 (PMC13205464; doi:10.2196/87898)
Supplement: Multimedia Appendix 2 [file nursing-v9-e87898-s002.pdf]

Table S2. Characteristics of Interventions

Author(s)Year, Country	Modalities and type of simulation	Security aspects covered	Direction of Effect	Type of intervention in the study	CASE
Breen et al.2019 Ireland .[47]	HFS-HF	Communication between professionals	Favors competency-based simulation	All groups 15 minutes training on the ISBAR tool. Group (E): HF room. Group (E+S) work in mixed discipline pairs: telephone calls on 4 standardized cases (3.5h). Group (E+PBP): same training as group E+S, reached competences	4 standardized clinical cases of acute patient deterioration
Son & Kim, 2019 Korea [36]	SP-HF	Communication with the patient	Favors simulation	CG: Prebriefing+ pretest (50 min),	Standardized pediatric scenario, with mothers of 5- year-old

				simulation 60 min, debriefing + post test (50 min) afterwards, and SEGUE-based communication (30 min). IG: SEGUE communication before	children admitted to the hospital for acute gastroenteritis with fever.
Jeong & Kim, 2020 Korea [37]	SP-HF	Falls and communication	Favors simulation	IG: 3 educational sessions on falls communication and SBAR method (theory, practice and discussion). CG: training according to guidelines on patient care and transfer of information.	Standardized patient falling out of bed

Liaw et al. 2020 China [49]	VR (simulated environment with avatar use) and live simulation (SP)-HF	Interprofessional communication and teamwork	Non-inferior (VR vs live)	All groups received 3 hours of team training on nurse-physician communication. IG: virtual environment and avatar, CG: SP. Each scenario lasted approximately 15-20 minutes and was followed by a 30-minute facilitator briefing	Sepsis and septic shock scenario.
Sanko & McKay ,2020 USA [33]	Board simulation (board game) to teach and develop systematic thinking-LF	Notification of incidents and adverse effects. System failures	Favors simulation	G C: Simulation scenarios. GI: 2017 course, included a tabletop simulation" Friday	Emergency Care

				Night in the ER".	
Lee & Kim, 2020 Korea [34]	HFS (IAM)-HF	Interprofessional communication	Positive association	Before the scenario training on SBAR-R communication and election of the leader of each team. Development of the scenario in 2 times: before a call to a fictitious doctor and performance of the task in a team after receiving instructions by phone	Acute myocardial infarction emergency care
Wai et al. 2021 Korea [48]	HFS-HF	Teamwork	Both improved	IG: combined classroom + simulation. CG 1: online training	Predetermined critical case scenarios: chest pain and weakness in MMII.

				on team-based learning, individual test and later in group, with those who would solve the simulation. CG 2: clinical case simulation. All groups training on patient safety, human factors and communication.	
Musharyanti et al. 2021 Indonesia [38]	Role play and simulated patients (actors)-LF	Medication administration	Favors simulation	IG: 18 hour training sessions over 5 weeks, overview and training activity with a 4C/ID approach includin	Oral and intramuscular drug administration

				g: presentation of real cases, small group discussion, reflection and simulation of oral and intramuscular drug administration. CG: 2 weeks, overview, two lecture sessions and video playback, and post-testing.	
Duet al. (2021) China [39]	SP in high fidelity room-HF	Pressure ulcer prevention (UPP)	Favors simulation	CG: pressure ulcer training in the conventional classroom 90 min, IG: training through simulated clinical	Three clinical scenarios from admission, hospitalization and deterioration of the disease

				scenarios	
Lee & Lim, 2021 Korea [32]	HFS (respiratory)-HF	Communication in transfer	Favors simulation	Students were divided into groups of 3 to 4 for 120-minute sessions consisting of 50 minutes of theoretical training and 70 minutes of simulation-based training.	2 patients with respiratory problems with high-fidelity simulator
Craig et al. 2021 USA [40]	HFS with computer package, electronic medical records (identification wristbands, carts, barcodes, computerized	Medication administration	Favors simulation	4 weeks of simulation. IG: 1. low-fidelity simulation on medication administration. 2. high fidelity simulation focused on safe medication administ	Scenario for administration of oral and subcutaneous medication (insulin aspart)

	records)			ration. 3. Clinical rotation. 4. high fidelity simulation + debriefing. CG: 1. standard training. 2 clinical rotation and 3-4 same as IG	
Raurell-Torreda et al. 2021 Spain [50]	Role playing SBAR.-LF	Interprofessional communication	Favors simulation	IG: divided into subgroups of 20 students for 1 hour role play session, learning objectives focused on basic professional health care skills, teamwork, use of SBAR worksheet and role distribution in	Patient in shock in an emergency department setting (based on a clinical case from the National League for Nursing) to assess teamwork and non-technical skills.

				respiratory tract management, nursing procedures and techniques, use of documentation. Patient assessment and intervention in 3 nursing roles: procedures, assessment and follow up.	
Park & Kim, 2021 Korea [41]	HFS (mannequin)-HF	Systemic and organizational factors. Diagnostic errors	Favors simulation	IG: The PDS-IB, and CG: simple-PDS. The scenario theme for the simulations in both groups was patient deterioration. The simulati	Patient with chronic obstructive pulmonary disease transferred from the emergency room to the inpatient ward (worsening).

				ons for both groups consisted of 1.5 h	
Chen et al. 2022 Taiwan [51]	SP-HF	Teamwork	Comparable gains	In group 1 (received IPE training - followed by EPS) and group 2 (received EPS training followed by IPE training). Simulation training was structured for 4 weeks (3 h per week) that incorporated a 2- week IPE program during which medical and nursing students were trained together	Critically ill patients with American Heart Association (AHA) guidelines for cardiopulmonary resuscitation and emergency cardiac care.

Pol-Castañeda et al. 2022 Spain [42]	SP (clinical case)-HF	Medications	Favors simulation	Briefing, simulation scenarios were conducted in 24 groups of 6 to 8 students, each playing a different role.	3 scenarios: hypocalcemia due to gastrointestinal disease in the emergency department, respiratory infection, paracentesis
Goldsworthy et al. 2022 Canada England Scotland and Australia [44]	VR-HF	Patient deterioration	Favors virtual simulation	The treatment group completed six VR of medical surgical nursing case studies over three weeks (two per week). Two VR were completed each week that they could repeat.	Acute deterioration care: angina/cardiac arrest; anaphylaxis; acute asthma exacerbation; COPD/pneumothorax, pulmonary embolism; and blood transfusion reaction.
Li et al. 2023 China [43]	HFS (standardized patient +mannequin)-HF	Patient safety, medication errors and adverse effects	Favors combined training	All: On-line course-training adverse effects, types, effects,	Scenarios of care in respiratory infection (medication) and hemiplegia (basic care)

				and communication and teamwork. IG:2 simulation cases in addition to training. CG: online training only	
Haerling et al. 2023 USA [45]	HFS mannequin and VR displays-HF	Safety risks. Interprofessionall and patient communication Medication Administration	Manikin superior/equivalent	Clinical experience: 4 hours of traditional clinical experience Mannequin simulation: simulation activities with mannequins in two scenarios VR were similar to that of the mannequin-based simulations.	Postoperative discharge care and postoperative emergency room readmission

				The groups varied according to the type of experiential learning activity they completed first	
Chou et al. 2024 Taiwan [46]	VR-HF	Communication	Favors VR simulation	IG received a VR training in nurse-patient communication skills two weeks prior to practice. The program was delivered in 4 sessions for 30 minutes each time for two weeks.CG received the 30-minute nurse-patient communication	Simulated hospital ward scenarios with four learning tasks: self-presentation, establishing a nurse-patient relationship, interaction, and medical history collection.

				teaching video that could be downloaded and viewed.	
Heier et al. 2024 Germany [35]	SP-HF	Notification of medication errors	Favors simulation	Interprofessional communication skills training on acute care medical errors (IG) with a cohort that did not receive interprofessional training (CG).	3 scenarios reported in a critical incident reporting system focused on medication errors caused by a chain of errors. Chemotherapy, wrong antibiotic and chemotherapy preparation with errors.

Note: 4C/ID: Four Components Instructional Design; AHA: American Heart Association; CG: Control Group; CUS: Concerned-Uncomfortable-Safety issue; EMR: Electronic Medical Records; EPS/SPE: Single Profession Education; HF: High Fidelity; HFS: Hight Fidelity simulator; IAM: High Fidelity Simulator for Acute Myocardial Infarction; IG: Intervention Group; IPE: Interprofessional Education; ISBAR: Identity-Situation-Background-Assessment-Recommendation; LF: Low Fidelity; MMII: Lower Limbs; PBP: Performance-Based Progression; PDS-IB: Patient Deterioration Simulation with Inattentional Blindness; QA: Quality Assurance; SBAR: Situation-Background-Assessment-Recommendation; SBAR-R: SBAR with Readback/Response; SEGUE: Set the stage, Elicit information, Give information, Understand patient perspective, End encounter; SP: Standardized patient; TeamSTEPPS: Team Strategies and Tools to Enhance Performance and Patient Safety; UPP: Pressure Ulcer Prevention; VR: Virtual Reality
