# Supplementary material for: Simulation-Based Training for Nursing Students to Improve Patient Safety: Systematic Review
Source: JMIR Nurs. 2026 May 26;9:e87898. doi: 10.2196/87898 (PMC13205464; doi:10.2196/87898)
Supplement: Multimedia Appendix 3 [file nursing-v9-e87898-s003.pdf]

JBI CHECK LIST FOR ANALITICAL CROSS SECTIONAL STUDIES 2020*

STUDY	Q1	Q2	Q3	Q4	Q5	Q6	Q7	Q8
Lee KR, Kim EJ.Korea 2020	Y	Y	Y	Y	U	U	Y	Y

1. Were the criteria for inclusion in the sample clearly defined
2. Were the study subjects and the setting described in detail
3. Was the exposure measured in a valid and reliable way?
4. Were objective, standard criteria used for measurement of the condition?
5. Were confounding factors identified?
6. Were strategies to deal with confounding factors stated?
7. Were the outcomes measured in a valid and reliable way?
8. Was appropriate statistical analysis used?

Options: YES(Y)-NOT(N)-UNCLEAR(U)

*Moola S, Munn Z, Tufanaru C, Aromataris E, Sears K, Sfetcu R, Currie M, Qureshi R, Mattis P, Lisy K, Mu P-F. Chapter 7: Systematic reviews of etiology and risk . In: Aromataris E, Munn Z (Editors). *JBI Manual for Evidence Synthesis*. JBI, 2020. Available from https://synthesismanual.jbi.global
