# Supplementary material for: Simulation-Based Training for Nursing Students to Improve Patient Safety: Systematic Review
Source: JMIR Nurs. 2026 May 26;9:e87898. doi: 10.2196/87898 (PMC13205464; doi:10.2196/87898)
Supplement: Multimedia Appendix 4 [file nursing-v9-e87898-s004.pdf]

JBI CHECKLIST FOR QUASI-EXPERIMENTAL STUDIES 2023**

	Q1	Q2	Q3	Q4	Q5	Q6	Q7	Q8	Q9
Son HK, Hee Kim D. Korea, 2020	Y	Y	Y	Y	Y	Y	Y	Y	Y
Wai AKC, Lam VSF, Ng ZLH, et al. Korea, 2020	Y	Y	Y	N	Y	Y	Y	Y	Y
Musharyanti L, Haryanti F, Claramita M. Indonesia, 2021	Y	Y	Y	N	N	Y	Y	Y	Y
Lee DH, Lim EJ. Korea, 2021	Y	N	N/A	N/A	Y	N/A	Y	Y	Y
Craig SJ, Kastello JC, Cieslowski BJ, Rovnyak V.USA , 2021	Y	Y	Y	Y	Y	Y	Y	Y	Y
Chen HW, O'Donnell JM, Chiu YJ, et al. Taiwan, 2022	Y	Y	Y	Y	Y	Y	Y	Y	Y
Pol-Castañeda S, Carrero-Planells A, Moreno-Mulet C. Spain, 2022	Y	N	N/A	N/A	Y	N/A	Y	Y	Y
Goldsworthy S, Muir N, Baron S, et al Canadá, Inglaterra, Escocia, Australia, 2022	Y	N	N/A	N/A	Y	N/A	Y	Y	Y
Li H, Zhao W, Li B, Li Y. China, 2023	Y	Y	Y	U	N	Y	Y	Y	Y
Haerling K, Kmail Z, Buckingham A. USA, 2023	Y	Y	Y	Y	Y	Y	Y	Y	Y
Heier L, Schellenberger B, Schippers A, Nies S, Geiser F, Ernstmann N. Germany, 2024	Y	Y	U	Y	Y	Y	Y	Y	Y

Options: YES(Y)-NOT(N)-UNCLEAR(U)-N/A(not aplicable)

1. Is it clear in the study what is the “cause” and what is the “effect” (i.e. there is no confusion about which variable comes first)?
2. Was there a control group?
3. Were participants included in any comparisons similar?
4. Were the participants included in any comparisons receiving similar treatment/care, other than the exposure or intervention of interest?
5. Were there multiple measurements of the outcome, both pre and post the intervention/exposure?
6. Were the outcomes of participants included in any comparisons measured in the same way?
7. Were outcomes measured in a reliable way?
8. Was follow-up complete and if not, were differences between groups in terms of their follow-up adequately described and analyzed?
9. Was appropriate statistical analysis used?

**Barker TH, Habibi N, Aromataris E, Stone JC, Leonardi-Bee J, Sears K, et al. The revised JBI critical appraisal tool for the assessment of risk of bias quasi-experimental studies. JBI Evid Synth. 2024;22(3):378-88.
