# Supplementary material for: Simulation-Based Training for Nursing Students to Improve Patient Safety: Systematic Review
Source: JMIR Nurs. 2026 May 26;9:e87898. doi: 10.2196/87898 (PMC13205464; doi:10.2196/87898)
Supplement: Multimedia Appendix 5 [file nursing-v9-e87898-s005.pdf]

JBI CRITICAL APPRAISAL TOOL FOR ASSESSMENT OF RISK OF BIAS FOR RANDOMIZED CONTROLLED TRIALS 2023***

	Q1	Q2	Q3	Q4	Q5	Q6	Q7	Q8	Q9	Q10	Q11	Q12	Q13
Breen D, O'Brien S, McCarthy N, Gallagher A, Walshe N. Ireland, 2019	Y	Y	Y	Y	Y	Y	Y	Y	Y	Y	Y	Y	Y
Jeong JH, Kim EJ.Korea, 2020	U	Y	Y	Y	Y	Y	Y	Y	U	Y	Y	Y	U
Liaw SY, Ooi SW, Rusli KDB, Lau TC, Tam WWS, Chua WL.China, 2020	Y	U	Y	N	Y	Y	Y	Y	Y	Y	Y	Y	Y
Sanko JS, McKay M.USA, 2020	N	N/A	Y	Y	U	Y	Y	Y	Y	Y	Y	Y	Y
Du YL, Ma CH, Liao YF, Wang L, Zhang Y, Niu G.China, 2021	Y	U	Y	N	Y	Y	Y	Y	Y	Y	Y	Y	Y
Raurell-Torreda M, Rascon-Hernan C, Malagon-Aguilera C, et al.2021, Spain	Y	Y	Y	Y	Y	Y	Y	Y	Y	Y	Y	Y	Y
Park J, Kim KJ.Korea, 2021	Y	Y	Y	Y	N	Y	Y	Y	Y	Y	Y	Y	Y
Chou CH, Tai HC, Chen SL Taiwan, 2024	Y	Y	Y	Y	N	Y	Y	Y	Y	Y	Y	Y	Y

Options: YES(Y)-NOT(N)-UNCLEAR(U)-N/A(not aplicable)

1. Was true randomization used for assignment of participants to treatment groups?
2. Was allocation to treatment groups concealed?
3. Were treatment groups similar at the baseline?
4. Were participants blind to treatment assignment?
5. Were those delivering the treatment blind to treatment assignment?
6. Were treatment groups treated identically other than the intervention of interest?
7. Were outcome assessors blind to treatment assignment?
8. Were outcomes measured in the same way for treatment groups?
9. Were outcomes measured in a reliable way
10. Was follow up complete and if not, were differences between groups in terms of their follow up adequately described and analysed?
11. Were participants analysed in the groups to which they were randomized?
12. Was appropriate statistical analysis used?
13. Was the trial design appropriate and any deviations from the standard RCT design (individual randomization, parallel groups) accounted for in the conduct and analysis of the trial?

***Barker TH, Stone JC, Sears K, Klugar M, Tufanaru C, Leonardi-Bee J, Aromataris E, Munn Z. The revised JBI critical appraisal tool for the assessment of risk of bias for randomized controlled trials. JBI Evidence Synthesis. 2023;21(3):494-506
